# Cytoreductive surgery ± hyperthermic intraperitoneal chemotherapy and the value of markers for acute kidney injury

**DOI:** 10.2478/abm-2024-0032

**Published:** 2024-12-16

**Authors:** Editorial Office of Asian Biomedicine

**Affiliations:** Faculty of Medicine, Chulalongkorn University, Bangkok 10330, Thailand

Systemic chemotherapy has been widely used for the treatment of advanced cancer with widespread metastasis [1]. In addition to chemotherapy, an enhanced recovery after surgery strategy has been studied and shown to considerably reduce complications, length of stay, and costs after most surgical procedures [2]. Cytoreductive surgery (CRS) ± hyperthermic intraperitoneal chemotherapy (HIPEC) has been proposed as a strategy to enhance recovery after surgery [2].

Systemic chemotherapy has been associated with acute kidney injury (AKI) through multiple pathophysiologic processes, including interstitial nephritis, inflammation, tumor lysis syndrome, and thrombotic microangiopathy [3]. Traditionally, a rise in serum creatinine (sCr) has been used to diagnose kidney function and has been incorporated into various definitions of AKI. However, there are several shortcomings of sCr due to its influence by various factors independent of kidney injury, e.g., muscle wasting, fluid overload, and malnutrition. Furthermore, a reduction in glomerular filtration rate detected by sCr is late and insensitive. Therefore, the reliance of AKI diagnosis based on sCr may delay the recognition and management of AKI.

Kidney biomarkers have been developed and validated in different patient populations. They vary by cell origins, physiological function, kinetics, and distribution. They may be freely filtered through the glomerulus, upregulated in kidney tubules, or released by inflammatory cells in response to kidney injury or stress [4]. These biomarkers can early predict or detect the onset of AKI. Furthermore, they may risk-stratify, prognosticate outcomes, and predict kidney recovery. Many of these kidney injury biomarkers have been approved by the United States Food and Drug Administration and European Medicines Agency, e.g., neutrophil gelatinase-associated lipocalin (NGAL), urinary insulin-like growth factor-binding protein 7, and tissue inhibitor of metalloproteinases-2 [5].

In the field of onconephrology, there are limited evidence of the utility of damage and functional kidney biomarkers with inconsistent results [6,7,8]. There have been no studies that evaluated kidney biomarkers in patients undergoing CRS ± HIPEC. Chen et al. [9] prospectively studied patients who received CRS ± HIPEC. They serially sampled urine NGAL, serum cystatin C, and serum β-2-microglobulin on the day before CRS ± HIPEC and then 2 h, 1 d, 2 d, 3 d, and 7 d after CRS ± HIPEC. The patients were treated for gastric cancer (36.0%), ovarian cancer (30.7%), and colorectal cancer (22.7%). The main indication for HIPEC was curative CRS ± HIPEC (69.3%). Only 13 patients with gastric cancer underwent adjuvant HIPEC. Most patients had previously undergone chemotherapy, and only 36.0% of the patients were chemotherapy-naive. There were no significant differences in any of the covariates between the AKI and non-AKI groups. They concluded that absolute measurements of these biomarkers cannot predict AKI. However, the heterogeneous characteristics of the patients, limited sample size, and low prevalence of AKI suggest that larger studies are indicated to support this finding.

Therefore, although CRS ± HIPEC has been proposed as a strategy to enhance recovery after surgery, more studies are needed to confirm the association with AKI. More importantly, changes in various biomarkers intraoperatively and postoperatively and their impact on guiding clinical decisions need to be explored.
